# CERP: Cooperative and Efficient Routing Protocol for Wireless Sensor Networks

**DOI:** 10.3390/s23218834

**Published:** 2023-10-30

**Authors:** Nesrine Atitallah, Omar Cheikhrouhou, Khaleel Mershad, Anis Koubaa, Fahima Hajjej

**Affiliations:** 1 Faculty of Computer Studies, Arab Open University, P.O. Box 84901, Riyadh 11681, Saudi Arabia; n.atitallah@arabou.edu.sa; 2 ISIMa Mahdia, University of Monastir, Monastir 5111, Tunisia; 3 CES Laboratory, National School of Engineers of Sfax, University of Sfax, Sfax 3038, Tunisia; 4 Robotics and Internet of Things Laboratory, Prince Sultan University, P.O. Box 66833, Riyadh 11586, Saudi Arabia; akoubaa@psu.edu.sa; 5 Computer Science and Mathematics Department, School of Arts and Sciences, Lebanese American University, Beirut 13-5053, Lebanon; khaleel.mershad@lau.edu.lb; 6 Department of Information Systems, College of Computer and Information Sciences, Princess Nourah bint Abdulrahman University, P.O. Box 84428, Riyadh 11671, Saudi Arabia; fshajjej@pnu.edu.sa

**Keywords:** Arduino-based sensor nodes, ContikiRPL, cooperative communications, RPL, routing, selective relaying

## Abstract

Wireless sensor networks (WSNs), constrained by limited resources, demand routing strategies that prioritize energy efficiency. The tactic of cooperative routing, which leverages the broadcast nature of wireless channels, has garnered attention for its capability to amplify routing efficacy. This manuscript introduces a power-conscious routing approach, tailored for resource-restricted WSNs. By exploiting cooperative communications, we introduce an innovative relay node selection technique within clustered networks, aiming to curtail energy usage while safeguarding data dependability. This inventive methodology has been amalgamated into the Routing Protocol for Low-Power and Lossy Networks (RPL), giving rise to the cooperative and efficient routing protocol (CERP). The devised CERP protocol pinpoints and selects the most efficacious relay node, ensuring that packet transmission is both energy-minimal and reliable. Performance evaluations were executed to substantiate the proposed strategy, and its practicality was examined using an Arduino-based sensor node and the Contiki operating system in real-world scenarios. The outcomes affirm the efficacy of the proposed strategy, outshining the standard RPL concerning reliability and energy conservation, enhancing RPL reliability by 10% and energy savings by 18%. This paper is posited to contribute to the evolution of power-conscious routing strategies for WSNs, crucial for prolonging sensor node battery longevity while sustaining dependable communication.

## 1. Introduction

Wireless sensor networks (WSNs) are an important part of the infrastructure that supports the Internet of Things (IoTs)  [1,2]. Due to the advancement of the underlying technology, WSNs have found widespread use in a variety of contexts [3]. As a result, they are extensively used in many different disciplines to conduct research, monitor the environment, monitor industry and even collect intelligence for defense and national security purposes [4,5]. The primary responsibilities of sensor nodes are detecting and communicating data correctly to the cluster head (CH). The latter is concerned with gathering data from nodes in the cluster and transmitting these to the hub [6]. But, the routing process is the one that uses the most power [7]. Therefore, it is crucial to make routing as efficient as possible to save energy and extend the lifetime of sensors. To address the requirements of WSNs, the Internet Engineering Task Force (IETF) has proposed the Routing Protocol for Low-Power and Lossy Networks (RPL) [8], which operates over IPv6 [9]. It is a powerful routing system that can be readily tweaked to suit the user’s needs by adjusting the weights of various objectives. While this is happening, RPL nodes may become unreliable as they choose routes that are less efficient than others [8]. Promoting RPL reliability by the selection of more stable and higher-quality routes remains an unresolved research challenge in RPL design. Several research efforts focus on improving the RPL protocol so that it can satisfy the stringent needs of WSNs in terms of energy savings, reliability, lifetime, latency, etc.

RPL reliability can be improved by leveraging the wireless medium’s broadcast capabilities to establish effective communication among its components. More precisely, channels from the source to the relay nodes, which have received a copy of the data packet, are utilized in every transmission strategy between any source–destination pair. This serves to instigate spatial diversity at the destination [10].

The recipient combines multiple signal duplicates, making use of the channel statistics established between itself and the different transmitters. In case of a transmission failure, a chosen relay is utilized to transmit the same data, providing a more energy-efficient alternative compared to a direct transmission from the source to the destination. Relay techniques become especially relevant when there is a significant distance, an extensive mid-range, or less-than-ideal channel conditions between the source and the destination. In this paper, we leverage this cooperative relay technique to enhance data transmission reliability and energy efficiency in an RPL protocol. Moreover, several studies performed on the subject of wireless communication were theoretical and have only been assessed via the use of simulation tools. In order to verify the efficacy of recently suggested methods in the wild, further test-bed trials are required. Our study departs from the aforementioned research by proposing *CERP*, which is based on the preferred relay selection rule and is implemented in an Arduino-based node. More precisely, the main contributions of this paper are:

1. **Novel relay selection algorithm**: We introduce a novel relay selection algorithm, seamlessly integrated into the ContikiRPL framework, which significantly enhances reliability and energy efficiency in wireless sensor networks (WSNs) through cooperative diversity.
2. **Cooperative-aided routing protocol (CERP)**: We develop the cooperative-aided routing protocol (CERP), a new routing protocol for WSNs. CERP is founded on our innovative relay selection method and the conventional RPL, demonstrating substantial improvements in routing performance.
3. **Practical implementation and evaluation**: We not only propose these advancements but also demonstrate their practical applicability. We map the CERP protocol onto an Arduino-based node equipped with an Xbee module and design a functional prototype using the Contiki operating system (OS) and Arduino Mega board. Moreover, we rigorously evaluate the CERP protocol’s performance using off-the-shelf Arduino-based nodes, establishing its real-world effectiveness.

The remainder of this paper is structured as follows. Section 2 gives an overview of the RPL routing mechanism. Section 3 discusses related works dealing with the enhancement of the RPL protocol. Section 4 provides a detailed formulation of the relay selection algorithm and presents the description of the proposed cooperative-aided routing protocol based on RPL. Section 5 presents the prototyping and the performance evaluation of the proposed protocol. Finally, Section 6 draws out concluding remarks.

## 2. RPL Routing Mechanism Overview

Numerous embedded devices with constrained power, memory, and computing capabilities are joined through various connections, such as IEEE 802.15.4, to form WSNs [1]. The IETF ROLL working group created an IPv6 RPL to help with battery life. It may be utilized in data-collecting networks while using very little power. Here, we provide an analysis of the RPL routing mechanism to expose its limitations, for which numerous research works have been conducted. Moreover, we summarize the abbreviations used throughout this paper in Table 1.

**Figure 1:**
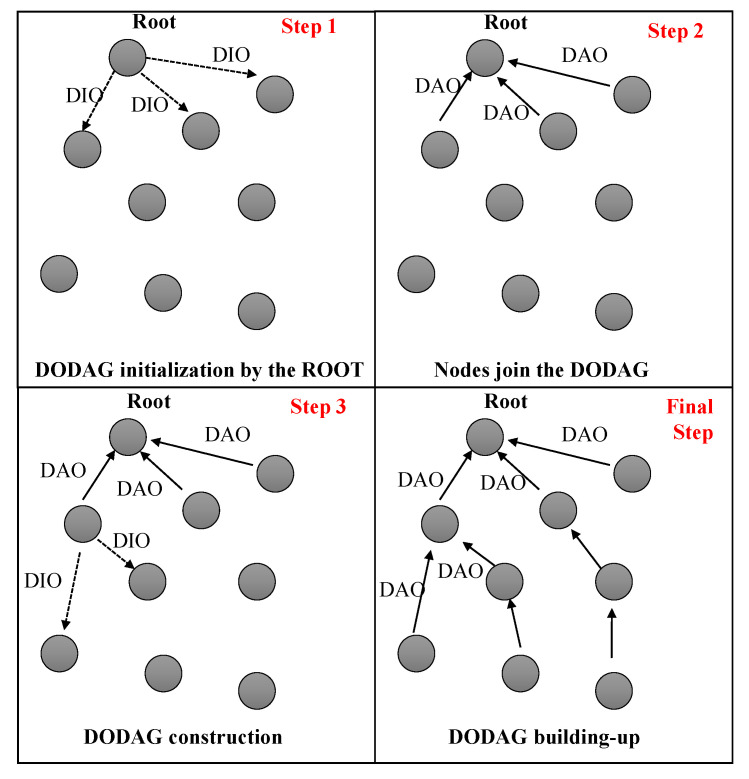
RPL DODAG building process.

Routing in RPL is performed using the destination-oriented directed acyclic graphs (DODAGs) idea, making it a distance vector routing protocol [11]. This is accomplished by constructing and updating a decentralized data structure whilst the WSN is in operation. The RPL method constructs this kind of distributed routing table to direct data packets throughout the network [12]. A root node is one that has no child nodes or outbound connections. The low power and lossy network is connected to the Internet and other external networks through “roots”. Using three predetermined messages, the root node chooses the optimal way based on predefined parameters (such as hop count, energy cost, dependability, latency, etc.) as shown in Figure 1:

- Destination information object (DIO): sent from the DODAG’s root to its child nodes in order to perform certain operations.
- Destination advertisement object (DAO): broadcasts information about a destination node up the distributed outage distribution graph (DODAG) to update the routing tables of parent nodes.
- DODAG information solicitation (DIS): in the case of grounding and floating DODAGs, it can help locate them. It is the DIO’s job to respond to a DIS transmission.

Depending on the specific use case, we categorize RPL functionality into one of three distinct modes [13] as follows:

1. **Collect protocol**: Data from all the other nodes are gathered via the collect protocol and then sent to the root node. As for the route, all one have to do is follow the DODAG.
2. **Distribute protocol**: With the help of the distribute protocol, information may be sent from the hub to individual nodes. The path is determined by using the name and origin place provided in the DAO message.
3. **Peer-to-peer protocol**: Data may be sent from one node to another via the P2P protocol, with the most often used route being through the parents or the root node.

In this paper, our focus is primarily placed on the collect mode, as our objective is to communicate detected leaks to the cluster head (CH) to facilitate apt responses. The initiation of constructing the RPL destination-oriented directed acyclic graph (DODAG) to back the collect protocol is spearheaded by the root node. DIO messages are multicast, reaching all accessible nodes. Each node’s rank value must be ascertained utilizing an objective function (OF), in alignment with RPL standards [14,15]. The OF specifies the process whereby RPL nodes rank input metrics, and hence how routes in a DODAG should be chosen and optimized [16]. Its primary function is to compute rankings according to predefined routing metrics including latency, throughput, and connectivity, as well as to define routing limits and optimization goals. As a result, developing the effective OF remains a topic of inquiry. After a node has received a DIO, it will begin counting down from that point. As soon as the timer runs out, they broadcast DIO messages to all of their nearby neighbors. This means that every node in the DODAG knows its stable set of parents and can reliably send packets down the route to the DODAG’s center, as shown in Figure 1.

Yet research shows that the packet loss ratio for multi-hop lines may approach 20 percent or more as the number of hops grows [8]. Thus, selecting more stable and high-quality routes is a viable method to improve RPL’s reliability. This is achieved by taking into account more efficient link quality [9]. In the coming sections, we will include numerous works and research that focus on improving the RPL routing mechanism for greater reliability.

## 3. Related Works

In this section, we discuss related papers dealing with RPL problems and highlight our contribution with regard to the literature. Much work has gone into creating new energy-efficient routing protocols and improving existing ones. In the realm of WSNs, RPL is a well-known example of an energy-efficient protocol. In addition, the Internet Engineering Task Force (IETF) has adopted it as the default standard routing protocol [17]. However, RPL nodes may be unreliable since inferior pathways are used. This is a topic for further study in RPL architecture since it might be used to improve the service’s dependability by allowing for the selection of more robust and high-quality routes [8]. In contrast, cooperative routing algorithms account for the potential for physical-layer cooperative transmission. In [18], the partner node’s relay selection for cooperative routing depends on criteria such as residual energy and SNR of the source–partner connection. The authors demonstrate that using SNR-based criteria yields superior outcomes regarding stability period, decreased latency, and packet loss. Optimal relay selection protocols that save energy and work together are presented for underwater WSNs. This uses the position and depth of the sensor nodes together to make its selections. In [19], the authors show that data packets are less vulnerable to variations in channel quality. They proposed CoopRPL, an implementation of RPL that incorporates a cooperative communication technique, to improve the dependability of advanced metering infrastructure (AMI) networks [20]. The authors in [21] addressed energy efficiency and demonstrated that cooperative methods may achieve better results than non-cooperative ones. In reality, if the energy is most effectively distributed between the source and the relay, the system performance of cooperative systems may be further enhanced. Many academics are interested in how cooperative diversity might be used to reduce power consumption in WSNs [21,22]. As cooperative transmission has become popular, it is considered in the routing mechanism, and there has been much effort put into developing and assessing cooperative routing protocols [23]. In [24], Matlab simulations are used to test and evaluate a suggested routing method that cooperatively considers energy route and channel awareness. Using the CoEPACA protocol, a higher degree of dependability may be attained with much less power. In [25], To ensure that IoT devices use as little energy as possible, a new routing measure, SPR, was created, and a more nuanced cross-layer goal function for RPL was suggested. Aslani et al. [20] demonstrated that, compared to RPL and opportunistic RPL, their suggested protocol improves the packet delivery ratio (PDR) by up to 20% and 10%, respectively, under best-effort conditions. They also demonstrated a 15% reduction in end-to-end latency compared to the RPL protocol.

In [26], the authors present the cooperation-aided routing protocol for lossy networks, which is the result of incorporating cooperative communication into the RPL protocol. Each node may send data to its desired parent through a relay node, which improves the dependability and decreases the energy usage at each hop in the network. The simulation findings show that their solutions may significantly reduce the energy use.

Similarly, in [27], considering the quality of the interlinks, the authors suggest a hybrid energy-efficient cluster-parent-based RPL routing protocol (HECRPL) to improve both efficiency and dependability. In terms of extending the network lifespan and delivering more consistent data transmission, they show via simulation results that their proposed protocol exceeds the benchmark RPL.

Energy efficiency has been investigated [28] from many directions, with some focusing on fostering cooperation between the nodes performing different sensing functions and others on creating numerous versions of the system. This resulted in the authors demonstrating a rise in node energy usage, complexity, and expense compared to RPL.

In addition, several publications investigated the RPL’s strengths and weaknesses, then provided suggestions for how it may be improved in the future so that it more closely mimics the actual world in [29]. In reality, we examine how well the contikiRPL implementation works in various network configurations. However, there are still many facets of RPL-enabled WSNs, such as signaling overhead, latency, and so on, that may be improved by more research and experimentation.

The authors in [30] assessed RPL’s performance across three parameters, namely network density, throughput, and sink localization. More precisely, three essential metrics are considered: Expected transmission count (ETX), hop count (HC), and energy. The evaluation results demonstrate that the parameters are influenced by the number of nodes in all scenarios. Notably, the ETX metric consistently exhibits a strong performance in terms of packet delivery ratio (PDR), while the energy metric consistently records the highest energy consumption among all the tested scenarios.

To address the challenge of reliability in RPL, the authors in [31] proposed RAARPL: reliability-aware adaptive RPL routing protocol. RAARPL enhances the RPL reliability by selecting parents based on multiple reliability-related criteria and considering path conditions during the decision-making process. This ensures network stability by controlling the parent selection and children assignment to minimize errors. Simulation results, compared to CLRPL and RPL protocols in various scenarios using Cooja, demonstrate the significant efficiency of RAARPL in improving data exchange reliability, successful delivery ratios, reducing topology instability, and enhancing network throughput.

Furthermore, some work addressed the security aspect of the RPL protocol [32,33,34,35]. In [36], the authors focused on the detection and mitigation of rank attacks within RPL. They proposed a rank attacks detection algorithm that minimizes the control packet overhead by appending extra fields in DAO and DIO messages and introduces a local alarm mechanism for energy conservation in cases of minor attack impact, alongside employing random sampling for efficient internal attacker identification. The authors in [37] introduced a novel RPL attack named dropped destination advertisement object (DDAO). The DDAO attack disrupts network connectivity by preventing the formation of downward routes, affecting a significant portion of the network. To counter this threat, the paper proposes an efficient, lightweight intrusion detection system that efficiently detects DDAO attacks through distributed monitoring of parent node behavior with respect to forwarded destination advertisement object (DAO) messages.

In summary, much effort must be put into studying, evaluating, and improving the RPL mechanism’s performances to increase its dependability and efficiency. With this in mind, we suggest a more robust version of RPL incorporating cooperative diversity into its routing mechanism to boost reliability and efficiency.

## 4. Cooperative Efficient Routing Protocol Formulation

This section outlines how to create a WSN-friendly version of ContikiRPL called CERP. The energy efficiency and reliability of a clustered network are both enhanced via the usage of cooperative diversity. Since the energy needed to transmit a signal from its source to its destination decreases with the square distance between them, using relay nodes as intermediary nodes is vital to this strategy. We will explain how to follow the rule that ensures that data arrive safely and quickly at the CH from sensor node. After solving an optimization problem in which the transmitted energy and the bit error probability (BEP) are considered the minimization criterion, the optimal route is selected. Afterwards, the innovative CERP is studied once its selection rule has been implemented in ContikiRPL.

### 4.1. Problem Formulation

In this subsection, we aim to choose the route between any source and the CH pair in the cluster that uses the least amount of energy [38]. This strategy utilizes the selective digital relaying (SDR) BEP criteria to reduce the transmission power consumption. To achieve this goal, BEP expressions are examined and developed for cooperative and non-cooperative communication channels [39]. In addition, the suggested optimization framework is described in great depth.

In this paper, we focus on a WSN organized in clusters. Within each cluster, whose *x* and *y* coordinates are known and the locations and distances between each other, we assume that *N* nodes are dispersed randomly.

**Figure 2:**
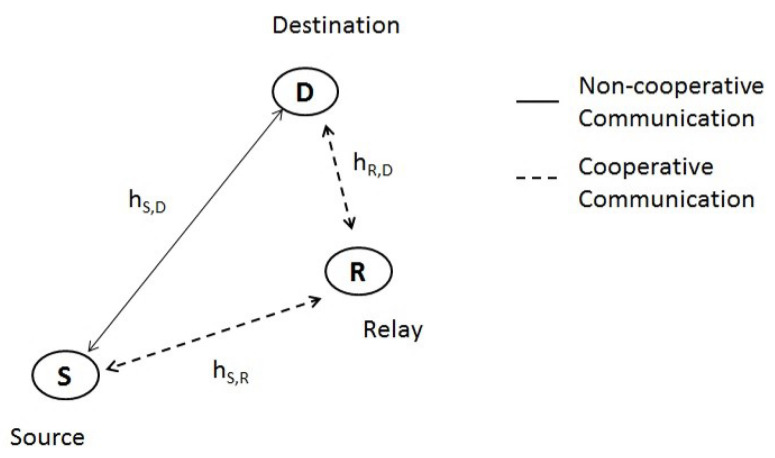
Different communication systems.

Suppose any node in a cluster wishes to communicate with the CH, which is a node selected by a clustering algorithm such as LEACH [40] that is responsible for collecting the information captured by the cluster sensors and delivering them to the center of maintenance and supervision. We denote the source by *S* and the destination by CH. Therefore, the signal goes via a series of connections before reaching its final goal. As illustrated in Figure 2, a relay *R* may take part by sending CH, a duplicate of the original signal across a two-hop connection (where required). Therefore, in the second stage, *R* may work together to relay the signal from the source to CH using either an amplify-and-forward (AF) or decode-and-forward (DF) method. Then, the maximum ratio combining (MRC) method [39] is used to combine the incoming signals at the CH. For a comprehensive channel model, we combine the path-loss with a temporal fading channel model (Rayleigh fading), as illustrated in Figure 2:

(1) $$Y_{S,CH}=h_{S,CH}X_{S}+W_{S,CH},$$

(2) $$Y_{R,CH}=h_{R,CH}X_{R}+W_{R,CH},$$

where $Y_{S,CH}$ ($Y_{R,CH}$) is the received signal at CH from the source (the relay resp.), $X_{S}$ ($X_{R}$) is the transmitted signal from *S* (*R* resp.), $W_{S,CH}$ ($W_{R,CH}$) is an additive and white Gaussian noise with variance $N_{0}$ and $h_{i,k}$ are the channel path loss-Rayleigh fading coefficients with $\left[h_{\left(i,k\right)}^{2}\right]=d_{i,k}^{-\alpha }$, with α is the path-loss exponent, [.] is the statistical average operator and $d_{i,k}$ is the distance between nodes in the cluster for i,k=0,…,N−1. Moreover, the signal’s power perturbed by Rayleigh fading is exponentially distributed. Hence, the resulting signal-to-noise ratio (SNR) is also exponentially distributed.

In this article, we draw a line between two distinct types of communication protocols, which are shown in Figure 2:

- Direct link transmission from a source node *S* to the CH is an example of non-cooperative communication. It is denoted by the symbol ${}_{d}$ and is referred to as direct transmission and noted ${}_{d}$;
- With the aid of a relay node *R*, a source node *S* may send data to the CH, and is referred to as cooperative communications, noted ${}_{c}$.

The following formula gives the BEP a direct communication system in which a source node *S* sends data to the destination node CH through a channel with a slow and frequency-flat Rayleigh fading coefficient [39]:

(3) $$Pe_{d}\left(\epsilon \right)=\frac{1}{2}\left(1-\sqrt{\frac{1}{1+\frac{1}{\sigma_{SCH}^{2}}}}\right),$$

where $\sigma_{SCH}^{2}$ is the mean of the SNR, noted $\gamma_{SCH}$, between source *S* and destination CH given by:

(4) $$f_{\gamma_{SCH}}\left(x\right)=\frac{1}{\sigma_{SCH}}\exp\left\{-\frac{x}{\sigma_{SCH}^{2}}\right\}\;\;\mathrm{where}\;\;\sigma_{SCH}^{2}=\frac{E_{d_{S}}^{b}}{d_{S,CH}^{\alpha }N_{0}}.$$

A generalized closed-form formulation of the BEP expressions for SDR systems is obtained and applied to a cooperative communication scheme in which a relay *R* is one of the N−2 nodes inside the cluster. To apply these expressions to our reduced-complexity model of the system [39], we have:

(5) $$\begin{array}{ccc} Pe(\epsilon |R_{sel}) & = & Pe_{d}\left(\epsilon |R_{sel}\right)Pe_{prop}\left(\epsilon |R_{sel}\right)\\ & + & \left(1-Pe_{d}\left(\epsilon |R_{sel}\right)\right)Pe_{coop}\left(\epsilon |R_{sel}\right), \end{array}$$

where

- $Pe_{prop}\left(\epsilon |R_{sel}\right)$ an error propagation occurs when the destination CH mixes the erroneously regenerated relay signal delivered by the chosen relay Rsel with the source signal.
- $Pe_{coop}\left(\epsilon |R_{sel}\right)$ an error occurs when the final destination CH wrongly combines the source signal with the appropriately regenerated relay signal delivered by the chosen relay Rsel.

According to [39], the probability of error propagation $Pe_{prop}\left(\epsilon |R_{sel}\right)$ can be approximated by the worst value $\frac{1}{2}$ whereas $Pe_{coop}\left(\epsilon |R_{sel}\right)$ is given by:

(6) $$\;Pe_{coop}\left(\epsilon |R_{sel}\right)=\frac{1}{2}\left(1-\frac{\sigma_{SCH}^{2}S_{SCH}}{\sigma_{SCH}^{2}-\sigma_{RCH}^{2}}-\frac{\sigma_{RCH}^{2}S_{RCH}}{\sigma_{RCH}^{2}-\sigma_{SCH}^{2}}\right),\;$$

where

$$\begin{array}{ccc} & S_{XY}=\sqrt{\frac{\sigma_{XY}^{2}}{1+\sigma_{XY}^{2}}}, & \\ \sigma_{SR}^{2}=\frac{E_{R}^{b}d_{R,CH}^{-3}}{N_{0}}, & \sigma_{SCH}^{2}=\frac{E_{S}^{b}d_{S,CH}^{-3}}{N_{0}}, & \sigma_{SCH}^{2}=\frac{E_{S}^{b}d_{S,CH}^{-3}}{N_{0}}. \end{array}$$

Bringing (6) and (3) into (5) yields the BEP expression for the SDR scheme:

(7) $$\;Pe\left(\epsilon |R_{sel}\right)\;=\;\frac{1}{2}-\frac{1+S_{SR}}{4(\sigma_{SCH}^{2}-\sigma_{RCH}^{2})}\;\left(\;\sigma_{SCH}^{2}S_{SCH}-\sigma_{RCH}^{2}S_{RCH}\;\right)\;\;,$$

where

(8) $$\begin{array}{cc} S_{SR}=\sqrt{\frac{1}{1+\frac{1}{\sigma_{SR}^{2}}}}, & S_{SCH}=\sqrt{\frac{1}{1+\frac{1}{\sigma_{SCH}^{2}}}},\\ S_{RCH}=\sqrt{\frac{1}{1+\frac{1}{\sigma_{RCH}^{2}}}}. \end{array}$$

The newly derived BEP given by (7) will be considered the basic constraint while selecting the preferred relay algorithm, which will be investigated in the following subsection.

### 4.2. Proposed Relay Selection Algorithm (PRSA)

**Figure 3:**
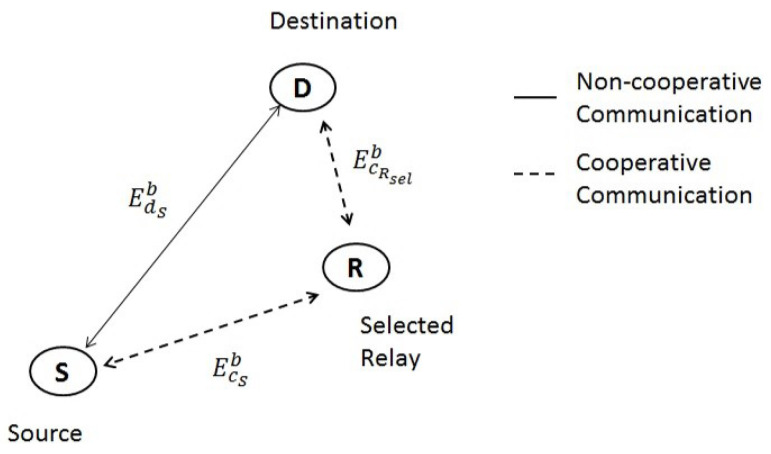
Respective power transmission links.

In this subsection, we will formulate the Algorithm of the selection of the optimal preferred relay that reduced the transmission power $E_{c_{R}}^{b}$ and $E_{c_{S}}^{b}$ (for *S* and *R*, respectively) while maintaining the BEP expressions given by (3) and (7) in the range of a predefined threshold value noted $P_{e_{th}}$ and satisfying the power constraint $E_{c_{R}}^{b}+E_{c_{S}}^{b}<E_{d_{S}}^{b}$ where $E_{c_{R}}^{b}$, $E_{c_{S}}^{b}$ and $E_{d_{S}}^{b}$ represent the required transmission power for each communication link, as schematized in Figure 3.

Essentially, we choose to keep the BEP below a certain threshold, denoted by $P_{e_{t}h}$, while minimizing the power sent by the transmitter. To do this, we use the cluster’s remaining N−2 sensor nodes as potential relays. We propose a method for determining the most efficient relay node by minimizing the sum of the transmission powers necessary for each communication link ($E_{c_{R}b}$, $E_{c_{S}b}$, and $E_{d_{S}b}$) while fulfilling the power constraint ($E_{c_{R}b}$, $E_{c_{S}b}$, and $E_{d_{S}b}$) as schematized in Figure 3.

The preferred relay is the one ensuring the minimum $E_{c_{R}}^{b}+E_{c_{S}}^{b}$, under the constraint that $P_{e}\left(E_{R_{i},b};E_{S,b}\right)$ is in the range of $P_{e_{th}}$. This can be formulated as follows:

(9) $$\begin{array}{c} R_{pre}=\underset{i\in R}{\arg\;\min\;}E_{c_{R_{i}}}^{b}+E_{c_{S}}^{b}<E_{d_{S}}^{b}\\ s.c\;P_{e}\left(E_{R_{i},b};E_{S,b}\right)\approx P_{e_{th}} \end{array}$$

where $R_{pre}$ denotes the preferred relay, $R=\{R_{1},R_{2},\cdots R_{N_{r}}\}$ where $N_{r}$ is the number of reliable nodes having source-node SNRs that exceed a predefined threshold $\gamma_{th}$. This optimization problem is solved numerically using the exact algorithm.

The main steps of the preferred relay selection algorithm are explained in the Algorithm 1. We highlight that cooperation is not always more energy-efficient than direct transmission. If $E_{c_{R_{i}}}^{b}+E_{c_{S}}^{b}>E_{d_{S}}^{b}$, this means that the direct (in this case, the CH will be set as a preferred relay in the routing table of the sensor node) transmission ensures the minimum transmission power in the range of $P_{e_{th}}$. Therefore, in the transmission strategy, cooperation only occurs when the constraint in (9) is verified. Otherwise, if $E_{c_{R_{i}}}^{b}+E_{c_{S}}^{b}<E_{d_{S}}^{b}$, the transmission from *S* to CH is performed via the selected relay only and no direct transmission link is considered. In this case, the proposed transmission strategy will not only increase the system’s energy saving but also promote communication reliability.
**Algorithm 1**  Preferred Relay Selection Algorithm
Compute the value of $E_{d_{S}}^{b}$ that maintains $P_{e_{th}}$ according to Equation (3) Find the set of reliable relays $R=\{R_{1},R_{2},\cdots R_{Nr}\}$ **for** each reliable relay $R_{i}\in R$ **do** Compute the values of $E_{c_{R_{i}}}^{b}$ and $E_{c_{S}}^{b}$ that maintains $P_{e_{th}}$ according to Equation (7) **if** $E_{c_{R_{i}}}^{b}+E_{c_{S}}^{b}<E_{d_{S}}^{b}$ **then**  Cooperative Transmission via relay $R_{i}$ **else**  Direct Transmission **end if** **end for**


### 4.3. CERP Description

**Figure 4:**
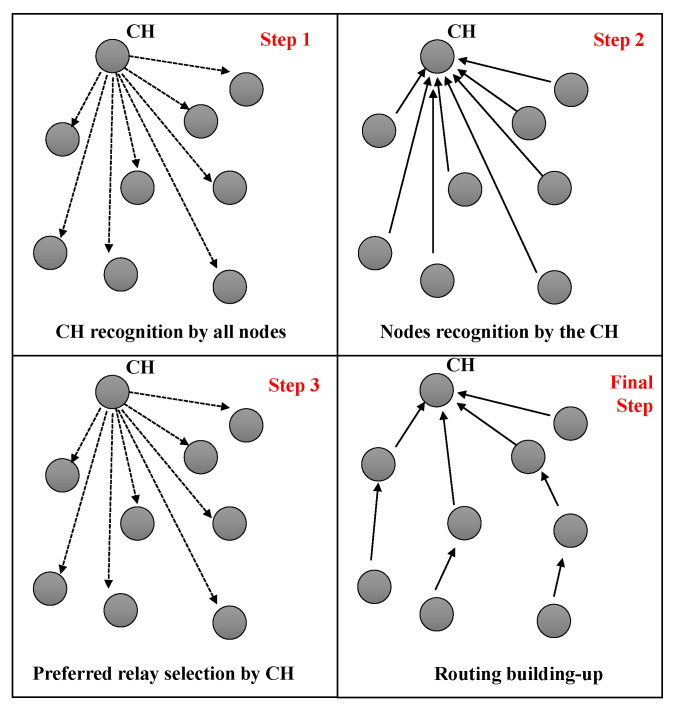
CERP construction steps.

The prevailing research on cooperative transmission strategies predominantly emphasizes physical layer approaches. Thus, to fully maximize the available bandwidth and power efficiency, recognizing the cooperative benefits at elevated levels, such as routing and security, is imperative. In this study, we propose to explore cooperative efficiency at both the lower network and application layers, introducing an RPL-inspired routing protocol, grounded in bit-power-transmission and bit error probability (BEP) within a cooperative-aided framework. It is requisite for each node in the network to maintain a routing table throughout the topology discovery phase, which encompasses a list of potential nodes that may ultimately interface with the cluster head (CH), identifying a selected node as the preferred relay. Furthermore, as a node dispatches a packet towards the CH, the CH, upon recognizing the sender, engages its preferred relay selection process to ascertain the packet’s subsequent destination. This suggests a prospect of enhanced reception reliability and reduced energy consumption with each transmission hop. The CH (or root node in the RPL procedure) instigates the network topology’s establishment. During the topology creation process, each network node furnishes its positional information to the CH. Upon the integration of a new node, the CH deduces the most fitting relay selection technique for it, taking into account the particular characteristics of the identified node. The power transmission of each link between the sensor node and the CH is computed during the recommended relay selection procedure. According to the energy link and packet loss as interpreted by BEP, the node facilitating the lowest transmission power, if present, is designated as the preferred relay (comparable to the preferred parent in the RPL framework). Consequently, the CH undertakes the responsibility of determining the subsequent hop for the source node, as shown in Figure 4.

Once the network has been discovered and its routing has been established, the CH should initiate a new topology creation process at regular intervals, an action denoted by the coherence interval, or $I_{c}oh$. Like ContikiRPL, the proposed protocol includes a neighbor-finding stage, denoted by [41]. During the relay selection algorithm phase, nodes also refresh their database of nearest neighbors. UDP was employed as the transport layer protocol. The contikiRPL selects a route for RPL routing based on the expected number of transmissions (ETX) needed to successfully transmit a packet across the connection [42]. The path that minimizes ETX from the source to the DODAG root is the route from a given node to the DODAG root. The routes, however, are constructed in the proposed protocol following PRSA using the BEP optimization as formulated in Equation (9). A brief comparison between RPL and the proposed protocol is summarized in Table 2.

**Figure 5:**
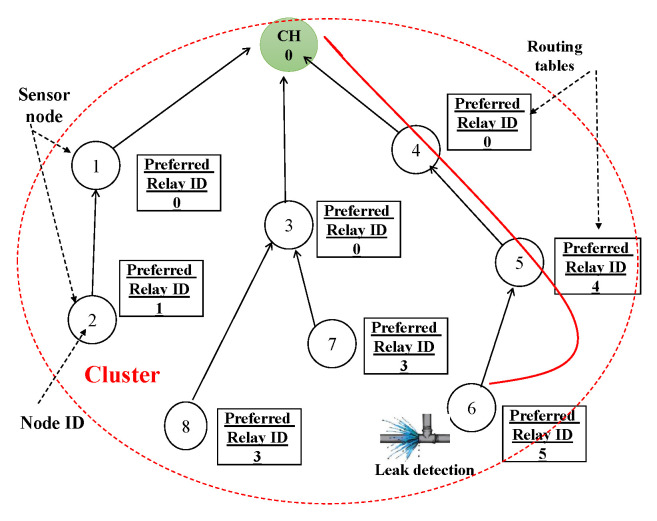
Example of leak detection in node 6 and the transmission alert to the CH.

To direct traffic upwards like that of an RPL DODAG, CERP simply requires the data acquired by PRSA. The PRSA reveals which relay the node prefers to use. When a node has to transmit data to the CH, it does so by first sending the data to the desired relay in the tree, which then forwards these to its preferred relay, and so on, until the data reaches the CH. In the case of a leak in the node, the paths leading to the CH are shown in Figure 5.

### 4.4. Routing Mechanism for Proposed Cooperative Efficient Routing Protocol (CERP)

CERP aspires to enhance network longevity and fortify its reliability as its paramount objective. The proposed protocol’s architecture unfolds in three pivotal steps, as elucidated in Figure 4:

1. Initial introduction and identification: in the initial phase, the cluster head (CH) presents itself to all other nodes within the cluster, identifying their neighbors. Although cluster formation is not the primary focus of this work and is presumed to pre-exist, each node submits its unique identifier to the CH for incorporation into the nodes matrix.
2. Execution of the preferred relay selection algorithm: detailed in Section 4.3, the CH calculates the preferred relay selection algorithm (PRSA) for each newly incorporated node $N_{i}$, where i=1⋯N−1. The aim is to discern the most optimal and energy-efficient next-hop for forthcoming transmissions, enabling node $N_{i}$ to attain a specific bit error probability (BEP), denoted by $P_{e_{t}h}$, while optimizing power utilization in its transmissions.
3. Construction of the routing table: the identifier of the chosen relay is stored in a node’s routing table, which is sustained by the node itself. Consequently, to transmit a packet to the CH, a node initially dispatches it to its favored relay within the tree. Subsequently, the packet is sent through a series of preferred relays until it reaches the CH.

As a further step, we will put the suggested protocol into practice on an Arduino-based SN and test it to ensure it works as intended while gauging its dependability and power consumption.

## 5. Prototyping and Performances Exploration

In this part, we will choose a realistic implementation of the proposed protocol and investigate its capabilities, contrasting them with the standard RPL. To do this, we shall describe the SN-level hardware in detail. Next, we will dive into how well the suggested protocol works in practice.

### 5.1. Design of the Proposed CERP

**Figure 6:**
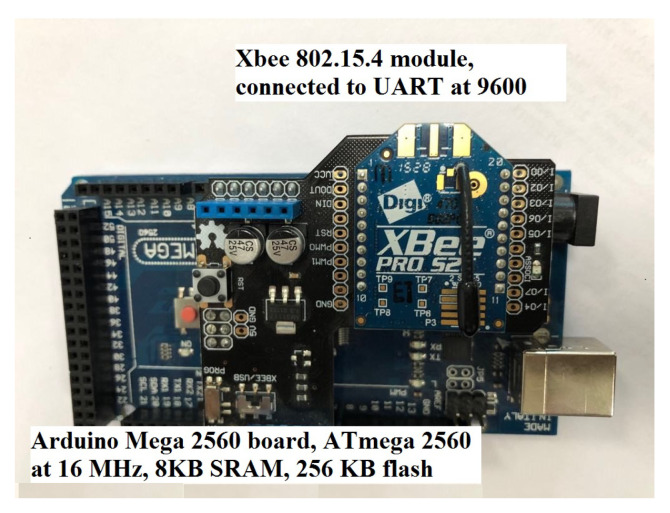
Sensor node built with the Arduino MEGA board.

A sensor node needs to be able to gather and share data on environmental characteristics. In addition, the hardware and software used to run it must minimize power consumption. Our team has deployed a working model of a leak-detecting system for water. The Arduino MEGA 2560 board (ADVANCE-TEC, Monastir, Tunisia), including an ATmega2560 operating at 16 MHz, is used to manage the pipeline and is one of the fastest Arduino boards available. This is also very typical of UART-based sensors like that in [43]. An XBee S2C pro-IEEE 802.15.4, (ADVANCE-TEC, Monastir, Tunisia) module by Digi [44] provides radio connectivity. As shown in Figure 6, the XBee module is linked to the Arduino through a UART operating at 9600 bauds to allow for bidirectional communication between the XBee module and the Mega board. Each sensor node, which is based on an Arduino, checks the flow rate against a standard. Following the suggested routing protocol, an alarm will be delivered to the CH when the water flow level exceeds the set threshold.

**Figure 7:**
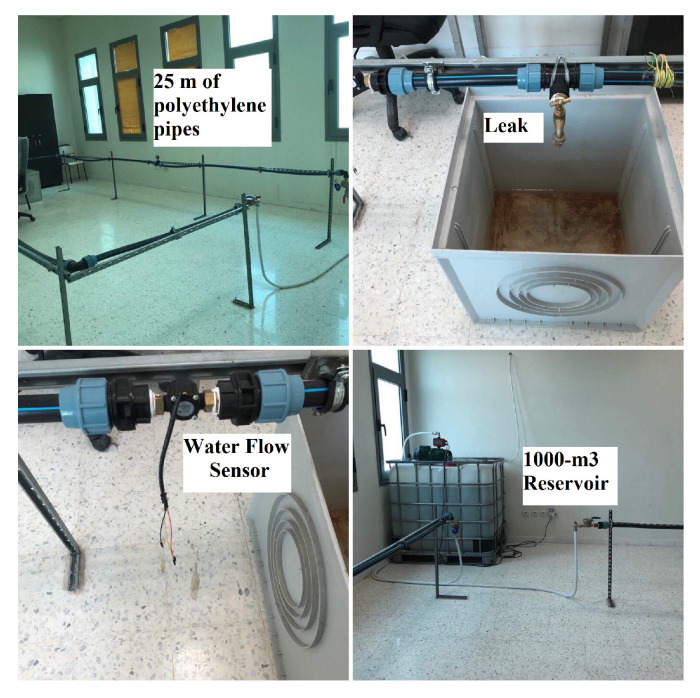
Real testbed demonstrator.

The sensors’ energy constraint requires using lightweight operating systems, such as TinyOS or ContikiOS, as explained in [8]. Due to its simplicity, flexibility, and greater availability, this work employs ContikiOS [45] as the default OS for physical deployment. Intriguing changes were made in the process of porting the code to Arduino. A demonstration is set up in the lab to assess the effectiveness of the suggested remedy. We test out the prototype in a functioning water pipeline. Indeed, putting the offered solution to work in the initial prototype is a foolproof method of guaranteeing its efficacy. We put this initial version through its paces in a working water pipeline demonstration specifically built for our needs, as illustrated in Figure 7.

### 5.2. Validation of the Proposed Prototyping

**Figure 8:**
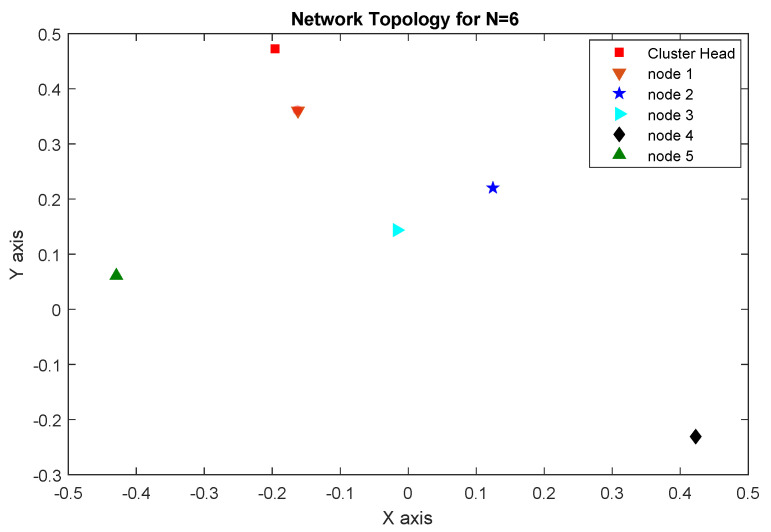
Network topology for N = 6.

**Figure 9:**
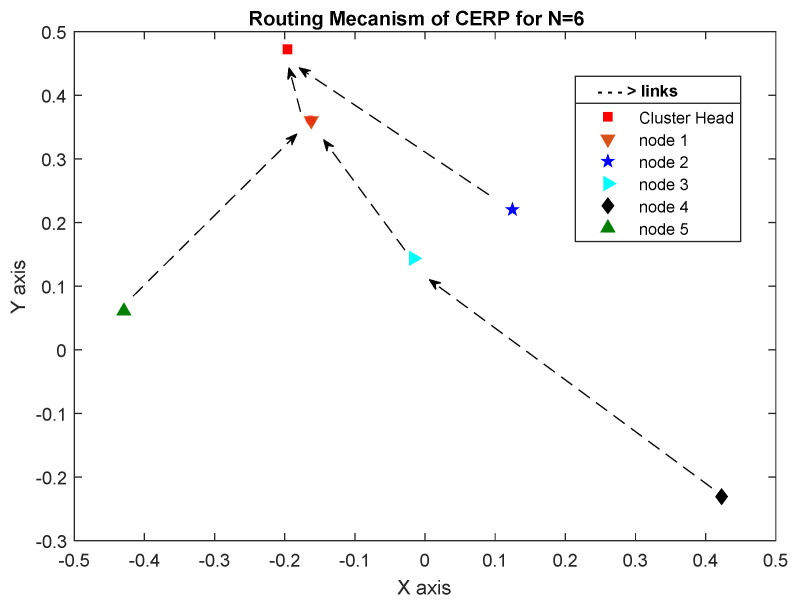
Routing construction for N = 6.

We will take into account a network of six Arduino-based SNs (one CH and five nodes) to ensure the suggested protocol is robust and reliable. According to Section 4.3, we divide the process into three “3” stages:

1. **Network topology stage**: At this point in the network’s development process, each proposed sensor node must initialize its network device, IPv6 stack, UDP, and data-transfer timer before it can begin sending data to the rest of the network. The CH can detect the sensor node immediately after startup. This work will focus on a cluster with six nodes, one of which will serve as the CH while the other five will be responsible for monitoring leak detection. Each node is based on the Arduino platform as described in Figure 6. The network topology is shown in Figure 8.
2. **Routing construction stage**: The CH is responsible for calculating the PRSA and determining the next hop for all nodes within the cluster. Thus, in the event of a leak, each sensor node is fully aware of the next-hop (preferred relay) IP address to connect with. When five nodes are added to the network, the routing structure shown in Figure 9 functions as intended. This second graphic shows us that there are three distinct ways for nodes to reach the CH: direct transmission (from nodes 1 and 2), two-hop transmission (from nodes 3 and 5), and three-hop transmission (from node 4).
3. **Packet delivery stage**: in this phase, if a sensor node detects a leak, it must immediately notify the CH. A sensor node’s preferred relay will forward the alert message to its preferred relay, and so on until the alert message reaches the CH. Future works will explain a practical implementation of the intended use case.

### 5.3. Power Consumption Evaluation

**Figure 10:**
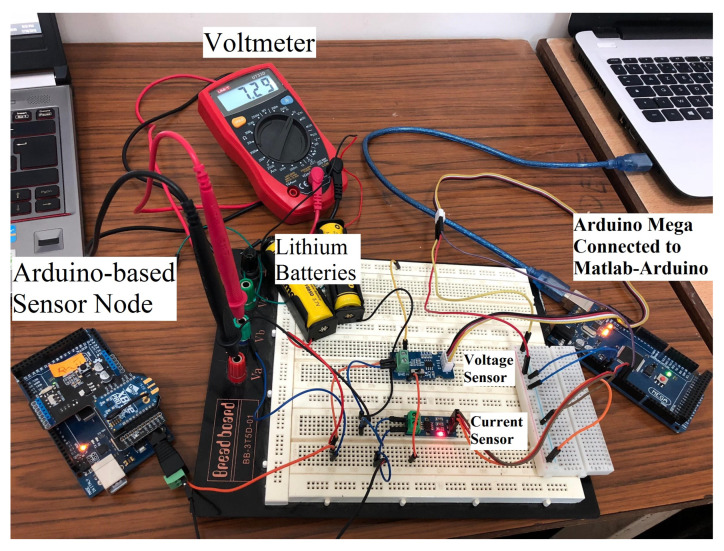
Experimental test.

To evaluate the power consumption of the CERP, the Arduino-based SN is interfacing with MATLAB using a free support package for the Arduino [46], as shown in Figure 10.

A voltage sensor, a current sensor (ACS712), and a voltmeter serve as our tools of choice for taking voltage and current readings. The sensor node is based on an Arduino and is powered by a pair of Lithium batteries, which can provide a voltage of up to 7 v. The support package handles reading the voltage value (or current) across the sensor attached to Arduino’s port A0 (or A3), allowing us to collect measurements and visualize them in real-time.

The power used by the CH is found by multiplying its voltage (Volt) by its current (Amps) for energy measurement:

(10) P=V×I(Watt).

Then, the energy consumed by the CH is the product of power (Watt) and time (hour) as follows:

(11) E=P×t(WattHour).

**Figure 11:**
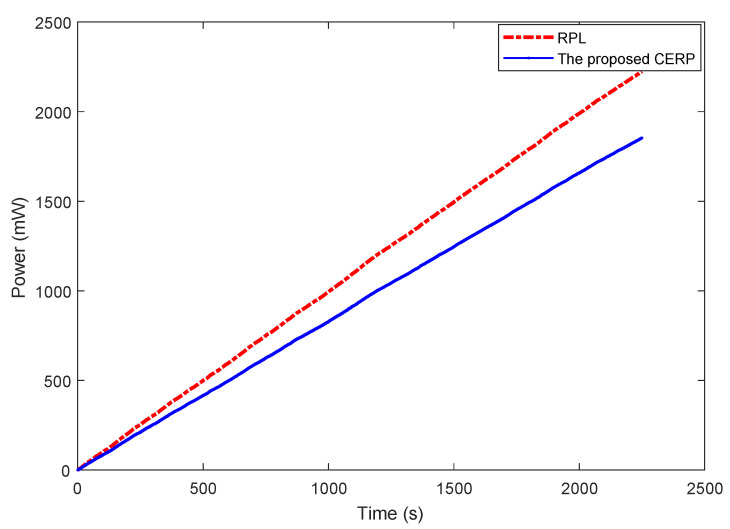
Comparison of the CH power consumption between the proposed protocol and RPL.

Thus, the energy consumed by the CH can be the instantaneous power (we use a sampling of 1 s for readings), as illustrated in Figure 11.

As depicted in Figure 11, the proposed protocol beats the RPL in terms of energy efficiency, and this is because, in the CERP mechanism, the CH chooses the ideal pathways that decrease the power transmission without compromising high-reliability levels. However, due to the extensive and ongoing nature of the RPL construction, the CH battery may be quickly depleted by the many exchanged DAO, DIS, and DIO messages.

### 5.4. Packet Loss Ratio

**Figure 12:**
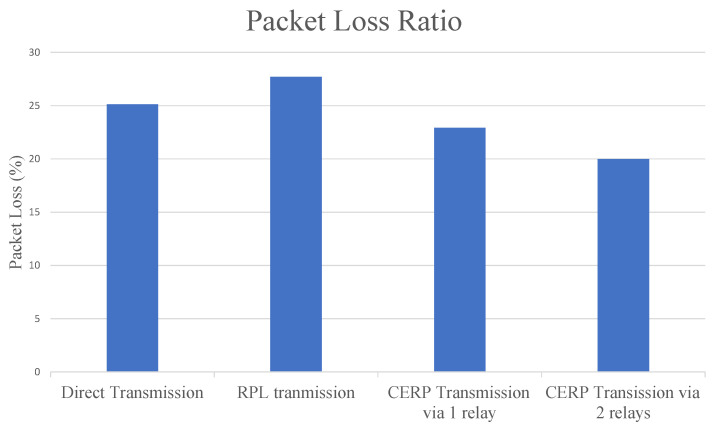
Comparison of the packet loss ratio between different transmission protocols for SNR = 15 dB, α=3 and data rate = 6.67 bps.

In the quest to assess the reliability of the proposed protocol CERP, we measure the packet loss ratio, defined as the number of lost packets to the total number of sent packets. As depicted in Figure 12, the proposed protocol outperforms the direct transmission and the RPL transmission [47] regarding reliability. Furthermore, we notice an improved level of reliability as the number of relays increases (i.e., as the number of hops increases). The selection of more reliable relays will lead to the building of reliable links where the predefined level of reliability is maintained.

### 5.5. Performance Evaluation of CERP

To evaluate the performance of our proposed protocol in terms of energy consumption, we compare our work with the related works, as discussed in Section 3. Table 3 summarizes the main results of the previous works when dealing with cooperative transmission and reveals the importance of this work where the routing protocol is bounded into an Arduino-based sensor node and tested not only by simulations but also by real tests. Table 3 shows the comparison of our proposed CERP with the main existing RPL improvement solutions. Some work only improves the reliability, such as [20,27,28], whilst others only improve energy [26]. However, our work improves both reliability and energy. Additionally, the existing work only evaluated their work by simulations. However, we evaluate our work by simulation and real test, and we found that real test results converge with the simulations ones. Improving RPL reliability will improve the packet delivery ratio. Moreover, energy saving will increase IoT devices and network lifetime.

As illustrated in Table 3, this work is the first mapped on an Arduino-based board. Furthermore, real tests demonstrate the effectiveness of the proposed protocol. All performance evaluations of the earlier solutions have been limited to the simulation analysis. Therefore, they ignored several practical aspects, such as mote limitations affecting real sensor mote implementation.

## 6. Conclusions and Future Works

This paper presents a novel routing protocol that utilizes an efficient and reliable rule to construct routes between every source–cluster head combination within the cluster. The proposed protocol aims to minimize the transmission energy consumption while maintaining a required level of dependability, resulting in reduced energy usage across the network. The proposed approach can significantly reduce the network’s overall energy consumption. To validate the protocol’s effectiveness, an Arduino-based sensor prototype was developed and tested in a real-world setting, demonstrating superior routing efficiency compared to the standard RPL. Moreover, the successful deployment of the prototype demonstrates the feasibility of our proposal in practical scenarios. The protocol’s efficacy is confirmed through experimental evaluations in a real-world setting, demonstrating superior efficiency and dependability compared to the standard RPL protocol. However, this study also highlights some limitations and open questions regarding IoT device connectivity, such as poor data transfer rates and storage capacities. Therefore, future research could investigate alternative hardware platforms with improved capabilities to address these challenges. The proof of concept presented in this paper provides valuable insights into the design and optimization of routing protocols in WSNs and their potential IoT applications.

In future works, we aim to develop more advanced energy-aware routing protocols that consider the dynamic nature of WSNs and their changing traffic patterns to further reduce energy consumption. We also aimed to extend the protocol to handle different types of traffic and data, including multimedia and real-time traffic, and evaluate its performance in these scenarios.

## Figures and Tables

**Table 1:** Abbreviations.

Abbreviation	Full Form
BEP	Bit error probability
CH	Cluster head
DAO	Destination advertisement object
DIO	Destination information object
DIS	DODAG information solicitation
DODAG	Destination-oriented directed acyclic Graphs
IETF	Internet Engineering Task Force
OF	Objective function
RPL	Routing Protocol for Low Power and Lossy Networks
RSSI	Received signal strength indication
SDR	Selective digital relaying
SNR	Relaying (SDR)
WSNs	Wireless sensor networks

**Table 2:** Comparison between RPL and CERP.

Protocol	RPL	CERP
Next hop	Preferred parent	Preferred relay
Building routes initialized by	Root	CH
Transport layer protocol	UDP	UDP
Network layer protocol	IPv6	IPv6
Control message type	ICMP	ICMP
Metric and constraint	ETX	$E_{d_{S}}^{b}$ + $P_{e_{th}}$

**Table 3:** Comparison between different works.

Refs.	RPL	Reliability	Energy
	**Improvements**	**Simulation Results**	
[20]	Incorporation of cooperative approach into the RPL	↑ 15%	–
[26]	Incorporation of cooperative approach into the RPL when transmitting data to the preferred parent	–	↓ 20%
[27]	Incorporation of hybrid energy-efficient cluster-parent based on RPL	↑16%	–
[28]	Incorporation of cooperative approach to create multiple instances among nodes	↑30%	–
		↑10%	↓ 30%
CERP	Cooperative approach to select routes	**Real-Test Results**	
		↑10%	↓ 18%

–: Results not provided.

## Data Availability

Not applicable.
